# Daidzein and puerarin synergistically suppress gastric cancer proliferation via STAT3/FAK pathway Inhibition

**DOI:** 10.1186/s41065-025-00419-2

**Published:** 2025-04-11

**Authors:** Jun Ge, Binguo Liu, Ling Ma, Jianyong Su, Ying Ding

**Affiliations:** 1 Department of Gastroenterology, Shanghai Municipal Eighth People’s Hospital, Shanghai, 200233 China; 2 Department of Pharmacy, No. 983 Hospital of the Chinese People’s Liberation Army, Tianjin, 300142 China; 3 Department of Gastroenterology, Yinchuan First People’s Hospital, Yinchuan Ningxia, 750001 China; 4 Department of Plastic Surgery, Shanghai Baoshan Hospital of Integrated Chinese and Western Medicine, Shanghai, 201900 China; 5 Department General Surgery, Shaanxi Provincial Rehabilitation Hospital, Xi’an Shaanxi, 710065 China

**Keywords:** Daidzein, Puerarin, Cell proliferation, Gastric cancer, STAT3/FAK pathway

## Abstract

**Background:**

Gastric cancer (GC) is the world’s health is seriously threatened by a prevalent form of aggressive tumor with a dismal prognosis. The occurrence of gastric cancer poses a concern for public health since it is a malignant tumor with an enhanced incidence and fatality level.

**Objective:**

The purpose of this study was to determine if the natural drug Daidzein (DZN) and Puerarin (PRN) together effectively suppress the proliferation of GC cells by blocking the STAT3/FAK intervention signalling pathways in BGC-823 cells.

**Materials and methods:**

Following a 24-hour treatment with the combination of DZN and PRN, the cells were examined for a number of assays. The MTT test was used to investigate the cytotoxicity of the DZN + PNR combination. Acridine orange/ethidium bromide (AO/EtBr) dual staining experiments were utilized to investigate apoptotic alterations, and Western blotting and flow cytometry were used to assess the protein expressions of the cell survival, cell cycle, proliferation, and apoptosis proteins.

**Results:**

Our findings showed that, DZN and PRN possessed anticancer properties by blocking the STAT3/FAK signaling cascade. Moreover, we discovered that the DZN and PRN combo reduced the protein levels of STAT3-FAK-dependent targeted genes, such as cyclin-D1, Bcl-2, Bax, MMP-2, prevented the phosphorylation and activation of STAT3, FAK.

**Conclusion:**

The current study’s findings suggest that the simultaneous administration of DZN and PNR can stop gastric cancer cells from proliferating, trigger apoptosis, and disrupt their cell cycle.

## Background

According to the GLOBOCAN investigations, cancer is the second most significant cause of mortality worldwide, with estimates of 19.3 million diagnosed and 10 million fatalities attributable to the disease in 2020 [1]. Globally, gastric cancer is one of the central causes of cancer-related mortality [2]. The occurrence of gastric cancer poses a concern for public health since it is a malignant tumor with an enhanced incidence and fatality level. About two-thirds of gastric cancer cases are diagnosed at an advanced stage, with early cases typically exhibiting no symptoms [3]. Many therapeutic approaches have been established in the last few decades, and chemotherapy has been demonstrated to be a key element of these approaches and can increase patients’ chances of survival when they have advanced gastric cancer [4]. Nevertheless, it has been documented that chemotherapeutic medication treatment is linked to serious side effects that could restrict its clinical application [5, 6]. In addition, several patients will experience distant metastases or localized recurrences even after radical resection.

According to the latest research, people who had gastric cancer (GC) had metastases into nearby and far-off tissues, including the lung, liver, peritoneum, and bone [7]. Therefore, it would appear that GC is a complex disorder, and managing this potentially fatal condition necessitates knowledge of the variables influencing its onset and malignancy. A substantial amount of statistics has been presented thus far regarding the variables causing the advancement of GC. GC malignancy’s most significant molecular mechanisms appear to be complicated [8]. GC therapy may be made more successful by identifying these possible avenues. Progressive gastric cancer has been effectively treated with molecular targeted therapies; however, the number of targeted medications available for clinical usage is relatively tiny [9]. For gastrointestinal cancer patients worldwide, target medications with high efficacy and minimal toxicity must be developed.

The signal transducer and activator of transcription (STAT) family of proteins is in charge of activating gene transcription and transferring extracellular signals from the cell exterior to the nuclei. Of the STAT protein family, only STAT3 expression is necessary to properly function specific tissues and organ systems. Amplification of STAT3 in the most of human malignancies is typically linked to a poor clinical outcome. STAT3’s gene transcription is implicated in multiple biological processes such as immune escape, angiogenesis, survival of cells, and proliferation [10].

To initiate a tyrosine phosphorylation cascade and attract Janus kinases (JAKs) to phosphorylate and stimulate JAKs, the aberrant signaling of classical STAT3 primarily dimers via the interaction of cytokines like IL-6 and their associated receptors on the cell surface. Rather, JAK activation phosphorylates particular receptor tyrosine residues, which are subsequently followed by a link with STAT3’s SH2 domain, which causes JAKs to phosphorylate STAT3 at Tyr705 in the membrane. When the phosphorylated Tyr705 site of STAT3 (p-STAT3) binds to the SH2 domain, it forms a homodimer and dissociates from the cell surface receptor. This causes STAT3 to move from the cytoplasm to the nucleus, further regulating gene level [11]. The STAT3 signaling system has been developed as a favorable goal for anti-tumor therapy due to its several effect pathways in tumor growth, metastasis, microenvironment development, and immunosuppression [12]. Thus, currently, one of the main areas of investigation for the creation of novel anticancer medications is focusing on blocking the STAT3 signaling system.

Apart from STAT3, aggressive tumor formation is also significantly influenced by focal adhesion kinase (FAK), an overemphasized tyrosine kinase in tumor cells that can govern cell survival, multiplying, and immigration [13]. Furthermore, the development and survival of tumor cells are markedly inhibited by the combined suppression of FAK and STAT3. Nevertheless, most drugs that solely target STAT3 or the FAK signaling cascade fall short of providing the optimal level of therapeutic efficacy [14]. In this instance, it is anticipated that novel therapies that simultaneously target FAK and STAT3 will increase the longevity of patients.

Creating compelling and targeted inhibitors of small molecules of the STAT3 signaling pathway has been complex and hotly contested. Finding novel small molecules in plant-based substances is one approach to creating novel medications [15]. Significant antibacterial and anticancer activities of certain natural compounds have led to their development as cancer treatment medications [16–19]. Numerous research studies have been published regarding the effectiveness of bioactive natural chemicals in treating gastric cancer [20]. Nevertheless, until now, research has solely examined natural goods as stand-alone treatments; no publications have examined the combined effects of using natural products. Thus, research assessing the synergistic effect of combining natural ingredients DZN and PRN was discussed in the present investigation.

**Scheme 1:**
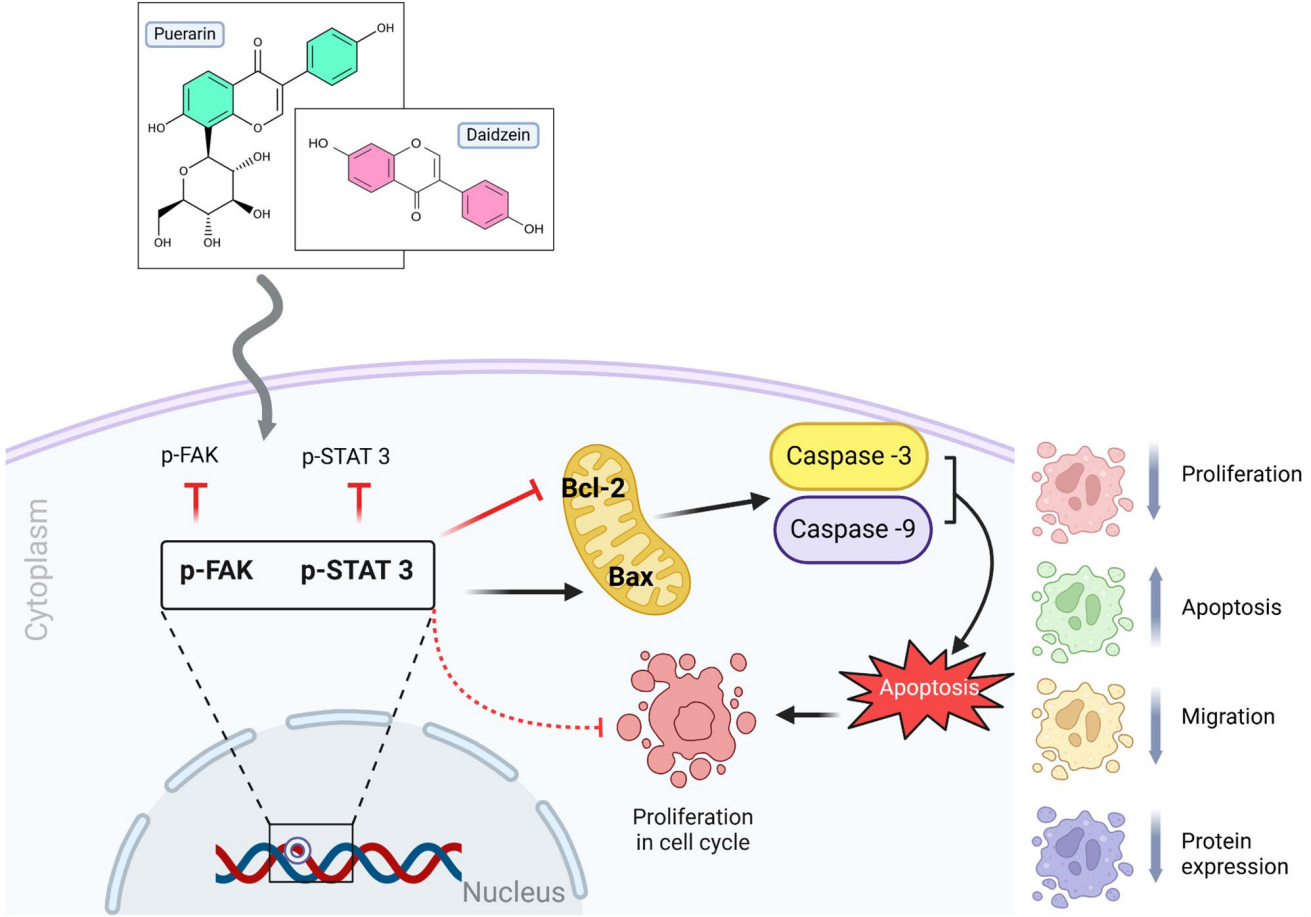
The anticancer properties of DZN and PRN as well as possible mechanisms underlying their effects on gastric cancer. We discovered that inhibiting STAT3/FAK signaling in gastric cancer by combining DZN and PRN a unique avenue investigation into cancer

The two main isoflavonoid chemicals extracted from kudzu are darzein and puerarin [21]. Plant-derived flavone 7,4-dihydroxyisoflavone, or daidzein, has been shown to have anticancer properties against various tumor types [22]. Nonetheless, daidzein’s anticancer effect has not been thoroughly studied, and its exact mechanisms still need to be determined. The primary isoflavone glycoside extracted from the conventional Chinese herb Radix *Pueraria lobate* is called purarin. Due to its numerous therapeutic qualities, including treating neurodegenerative illnesses and cardio-cerebrovascular disease, PRN has been used extensively. According to [23], puerarin can have anti-cancer properties and stop the proliferation of cancer cells. However, no scientific evidence supports that a combination of DZN and PNR has an anticancer effect on gastric cancer, particularly in target-specific pathways. To explore their potential synergistic chemopreventive effects against gastric cancer cells, DZN and PNR were combined in the current study. This research focused on the molecular processes involved in the STAT3/FAK pathways, which may indicate suppression at the early stages of gastric cancer development (Scheme 1).

## Methods

### Chemicals and reagents

Lonza Pvt Ltd. in China provided tetrazolium bromide (MTT), RPMI 1640 medium, phosphate-buffered saline (PBS), and fetal bovine serum (FBS combined with other components for cell culture. We purchased Daidzein (Catalog Number D7802), Puerarin (Catalog Number 82435) (> 98% purity), and Dimethyl Sulfoxide (DMSO) from Merck Sigma Aldrich Co. Ltd. in St. Louis, USA. The primary monoclonal antibodies, which included those for STAT3, FAK, MMP-2, Cyclin D1, Bcl-2, Bax, and β-actin, were supplied by Santa Cruz Biotechnology, USA. The chemicals were used precisely as received, without any further distillation, and deionized (DI) water was utilized for the whole process. All glassware was autoclaved before use.

### Cell culture and maintenance of gastric cancer cells

The Chinese Academy of Sciences’ Cell Bank (Shanghai, China) provided the human gastric cancer BGC-823 cell line, and human gastric epithelial cell lines (GES-1). The cells were grown in RPMI-1640 media that was enhanced with 10% FBS, 10 µg/mL streptomycin, and 100 U/mL penicillin. The cells were cultured in a humid condition at 37 °C with 5% CO_2_. Once the cell volume in a 175 cm^2^ flask achieved 80%, the cells were incubated with trypsin-EDTA for culturing and then rinsed with phosphate-buffered saline (PBS; pH 7.4). Every two to three days, the culture media was replenished.

### Evaluate for cell viability

Cell viability was evaluated using the MTT assay following treatment with various doses of DZN and PNR and specified dosages of the combined drugs (0, 10, 20, 30, 40, 50, and 60 µM). Each treatment has been tested through three replications. Trypsinized BGC-823 cells (1 × 10^4^ cells/well) were added to a 96-well plate, and left overnight for adhesion. After that, they received 24-hour therapy with three different groups such as DZN, PNR, and DZN + PNR. Then, each plate underwent another 4 hours of incubation at 37 °C with 20 µL of MTT added to each well. After being withdrawn from the MTT-containing media, the formazan crystals were added to each well and liquefied in 150 µL of DMSO. The plates were then agitated at 37 °C for 10 min. The colored solution’s 550 nm absorbance was then measured using a microplate reader. The cell viability was calculated using the formula shown below:

% cell viability = (OD value of test∕OD value of control) × 100.

### Evaluation for drug combined use

Drug analysis combination Interactions among drugs can be quantitatively assessed using the combination index (CI)-isobologram equation, where CI < 1 indicates synergism, CI = 1 indicates additive effects, and CI > 1 indicates antagonistic effects [24]. The software Compusyn© version 1.0 (ComboSyn, Inc. Paramus, NJ, USA) was utilized to produce the CI-effect plot, concentration-dependence curves, and concentration-effect assessment.

### Colony formation analyse

The colonization forming unit (CFU) assay assesses the cell line’s capacity to generate colonies from a single cell that includes control cells that were not treated sets and treatment groups that were administered different dosages of DZN, PNR, and DZN + PNR. For BGC-823 cell lines, cells at 80% diameter were separated and placed into 6-well plates in triplicate at 400 and 500 cells/well densities. The plates were allowed to incubate for 14 days under usual conditions and were checked daily to ensure colonies were growing. The media was withdrawn after 14 days, and the cells were stained for 15 min at room temperature using 0.5% (v/v) crystal violet (CV). The number of colonies was then calculated. Only colonies that could be seen were counted (diameters greater than 0.5 mm, no overlap) [25].

### Proliferation assessment by edu

The cells (1 × 104/well) received seeding in a plate with 96 wells before being exposed to DZN, PNR, and DZN + PNR for 24 h. The EdU proliferation test kit (RiboBio Co., Ltd, Guangzhou, China) was used to measure cell proliferation. A fluorescent microscope (Olympus IX51; Olympus Corporation, Tokyo, Japan) was used to see the stained cells.

### Morphological characterization of cell nuclei using Hoechst staining

To analyze the BGC-823 cells treated with DZN, PNR, and DZN + PNR, the nuclei were stained with Hoechst, and then fluorescence image analysis was performed. After 24 h of incubation, the BGC-823 cells were seeded at a density of 1 × 104 cells/mL on a sterilized sample. The cell structure was then stained with Hoechst and examined under a fluorescence microscope (Lawrence and Mayo India Pvt, Ltd.).

### Dual staining with AO/EtBr

AO/EtBr staining was used to determine the percentage of cell apoptosis in BGC-823 cells treated with DZN and PNR. In a nutshell, gastric cancer cells were plated (1 × 10^4^/well), and the cells were reared for 24 h at 37 °C. The cells were then given DZN, PNR DZN + PNR treatment and incubated for 24 h at 37 °C. Following the treatment stage, the treated cells were stained for 10 min with 100 µg/mL of AO/EtBr dye at a 1:1 ratio. Using a fluorescent microscope (3501, Lawrence and Mayo India Pvt, Ltd), the quantity of apoptotic cell death in the treated cells was measured.

### ROS generation

According to prior instructions, the ROS production test was carried out [26]. After being treated with DZN, PNR, and DZN + PNR for 24 h, the BGC-823 cells were rinsed with PBS and resuspended in culture media (without serum) containing 10 µM DCFHDA. Then, using flow cytometry, the generation of ROS was identified.

### Quantitative analysis of mitochondrial membrane potential (MMP)

The level of MMP in DZN, PNR, and DZN + PNR treated BGC-823 cells was measured using Rh-123 staining methods. To prepare this, 1 × 10^4^ BGC-823 cells were placed into each well of a 24 well plate, and the cells were subsequently treated for 24 h at 37 °C. The cells were then incubated at 37 °C for 24 h with DZN as well as PNR. Afterward, the cells were stained with Rh-123 at a dosage of 10 µg/mL for 30 min, and the brightness of the fluorescence was assessed using a fluorescence microscope (3501, Lawrence and Mayo India Pvt, Ltd.).

### Cell apoptosis assay by flow cytometry

BGC-823 cells (1 × 10^4^) were sown in every well and incubated for 24 h at 37 °C in the groups receiving treatment DZN, PNR, and DZN + PNR that received medium supplements. Three-fold dilute trypsin was used to extract BGC-823 cells, which were then twice-washed in PBS and re-suspended in binding buffer in an icy environment. The accumulation of FITC and PI completed the procedure, which was then detected using an apoptosis detection kit available commercially (Ebioscience, USA).

### Cell cycle arrest analysis

BGC-823 cells (1 × 10^4^) were seeded in each well. After 24 h of treatment with DZN, PNR, and DZN + PNR, the cells were trypsinized, recovered, and washed twice in ice-cold PBS. The cells were then fixed by adding 70% ice-cold ethanol, and incubated at 4°C overnight. Cells were then exposed to PI and ribonuclease A for 30 min. A flow cytometer was employed to count the number of cells at each step of the cell cycle.

### Western blot analysis

The impact of the DZN, PNR combo on the apoptotic and anti-apoptotic proteins in the experimental and control cells was investigated using Western blotting. In a 100-mm culture dish, BGC-823 cells were treated for 24 h with DZN, PNR, and DZN + PNR drug combinations. The media was removed, and the cells were repeatedly washed in PBS. The cells were then exposed to lysis buffer for more than 20 min. The proteins from the cells were then extracted using centrifugation from the supernatant. The bicinchoninic acid (BCA) protein assay kit (Pierce, Rockford, WI) was used to measure the concentrations of the total protein. From every concentration level of the sample, the same amount of protein was electrophoretically separated on a 12% SDS-polyacrylamide gel. Each transfer followed an hour of blocking the proteins with 10% skim milk in water before they were released. The primary antibodies against STAT3, FAK, MMP-2, Cyclin D1, Bcl-2, Bax, and β -actin were added at a v/v ratio 1:1000 after washing with PBS containing 0.1% Tween-20. The primary antibodies were removed by washing, and the secondary antibodies were added following an overnight incubation at 4 °C. After one hour of incubation at room temperature, the protein bands were discernible.

### Wound healing assay

Cells were seeded in a six-well plate at an average density of 2 × 10^5^ cells per well. When the cells achieved 90% confluence, a wound was created by scraping them with a pipette tip. The cells were exposed to varying doses of DZN and PNR. An inverted fluorescent microscope (Leica, Germany) captured DZN and PNR treatment photographs at 0 and 24 h, and the cell migration rate was estimated.

### Analysis of statistics

All the data was analysed with version 20 of the Statistical Package for Social Sciences (SPSS). The Shapiro-Wilk test was used to determine each test’s normality. Following a one-way ANOVA test on information with a normal distribution, Tukey’s HSD test was performed as a post-hoc analysis. The Kruskal-Wallis and Mann- Whitney non-parametric tests were used to assess data values that were not regularly generated. A significance level of *p* < 0.05 was applied to all data, which are displayed as the mean ± standard error (SEM).

## Results

### DZN, PNR impede the viability of gastric cancer cells

**Fig. 1:**
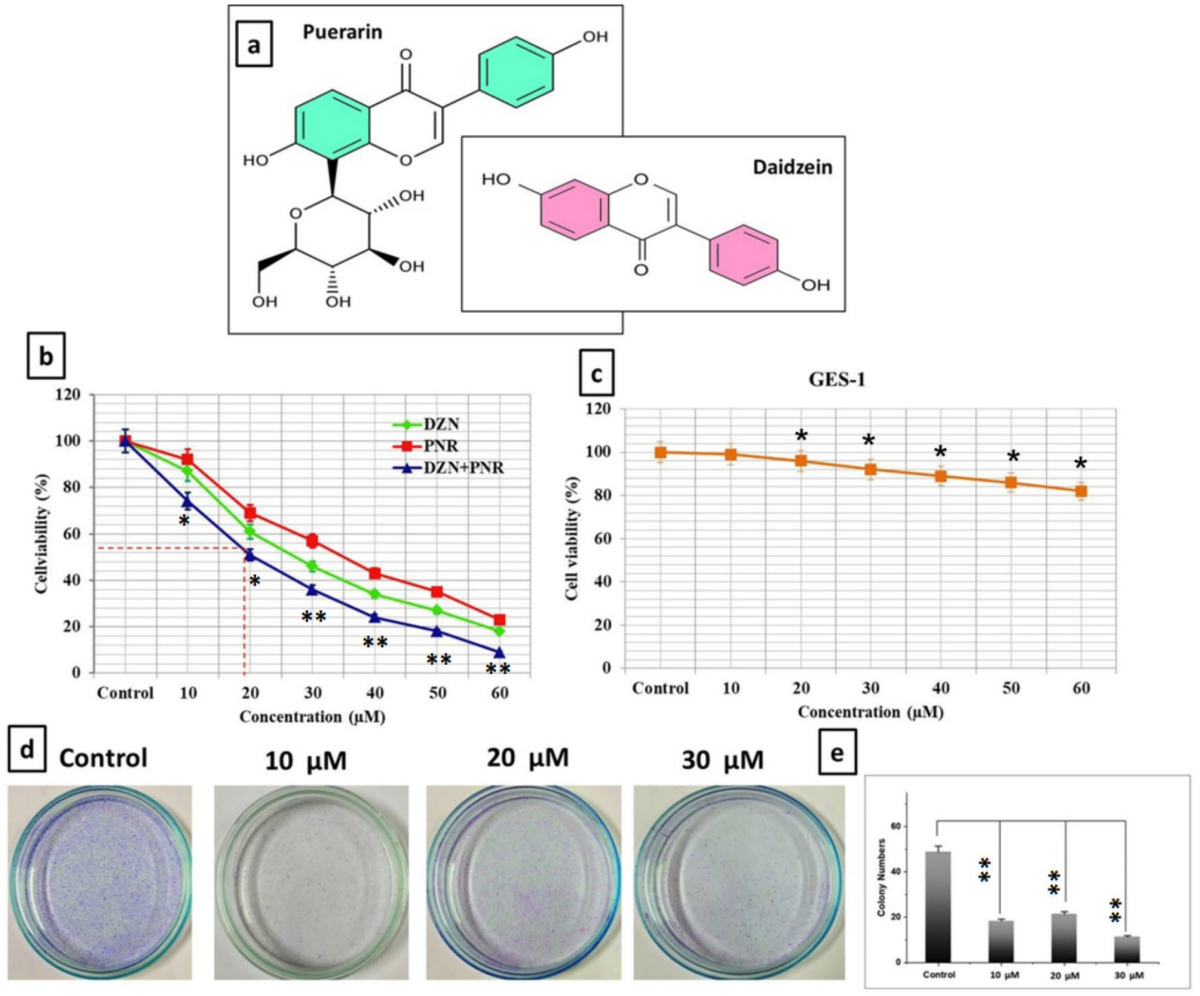
Puerarin and daidzein’s influence over BGC-823 cell growth. (**a**) The composition of daidzein and puerarin; (**b**) The MTT assays used to determine the inhibitory effects of various DZN and PNR doses on BGC-823 cell viability at 24 h. The outcomes demonstrated that DZN and PNR together had a dose-dependent inhibitory impact on BGC-823 cells. (**c**) GES-1 normal cell line for duration of twenty-four hours. The information is displayed as the mean ± SD of three different studies; in relation to the control group. (**d**) BGC-823 cells were unable to replicate when DZN and PNR were combined. (**d**) Typical pictures demonstrating how DZN + PNR (10, 20, and 30 µM) affects BGC-823 cells’ capability to form colonies after treatment. (**e**). BGC-823 cells’ related percentage of forming colonies after treatment is shown by bars (mean ± SD; *n* = 3). The data is shown as the mean ± standard deviation of three separate studies; *P < 0.05, **p < 0.01, relative to the control group

To evaluate the growth-inhibitory properties of daidzein and puerarin, their combined effects on the proliferating processes in the cells were studied at 24 h incubation (Fig. 1a). Figure 1 depicts the DZN and PNR components.

The present investigation initially looked at the cytotoxic properties of DZN, PNR, and DZN + PNR using the MTT assay in order confirm the impact of DZN + PNR on the proliferation of BGC-823 cells. For 24 h, the cells were grown with 0 − 60 µM DZN, PNR, and DZN + PNR together. The combined use of DZN + PNR treatment resulted in a considerable inhibition (**p < 0.01) of cell proliferation after 24 h (Fig. 1b). The proliferation of BGC-823 cells was significantly inhibited by combined use of DZN and PNR, as demonstrated in Fig. 1b. This effect was concentration-dependent, resulting in an IC_50_ value of 29.90 ± 1.03, 32.45 ± 1.21, (*p < 0.05) and 19.64 ± 0.71µM (**p < 0.01) for DZN, PNR, and the combination of DZN + PNR treatment for 24 h, respectively. The findings indicate that DZN + PNR has a strong anti-proliferation effect on human gastric carcinoma BGC-823 cells. In addition, Fig. 1c demonstrates no appreciable difference in cell viability when the combination of DZN + PNR treated GES-1 normal cells.

### Evaluate the colony formation

Furthermore, the combined treatment of DZN, PNR, and DZN + PNR related cell proliferation was investigated using the crystal violet test, which is depicted in Fig. 1d. After that, we treated BGC-823 cells with DZN, PNR, and DZN + PNR and conducted a colony formation experiment. The quantity of gastric cancer cell colonies treated with a combination of DZN + PNR (Fig. 1d, e) therapy was significantly reduced after 14 days of cell culture with DZN, PNR, or DZN + PNR. In conclusion, combining DZN and PNR can dramatically reduce the viability and proliferation of BGC-823 cells.

### Assessment of drug interactions

Using CompuSyn© software, we evaluated an integer DZN: PNR ratio (1:1) in order to compute the combination index (CI) and generate isobolograms. When two medications are used together, CI can be used to quantify their synergism or antagonism. A CI of 1 denotes an additive effect, while CIs of < 1 or > 1 denote synergism or antagonism. According to the combination index, five combos utilized in BGC-823 cells demonstrated a strong synergy of cell death. Table 1 shows the CI indices associated with certain combinations. The results demonstrated a dose-dependent trend, where CI values ranged from 0.52345 to 0.73498 across different concentrations (Table 1). At lower concentrations (10–30 µM), the combination exhibited a synergistic effect (CI < 1), indicating enhanced inhibitory activity compared to individual treatments. At higher concentrations (40–50 µM), the effect remained modestly synergistic (CI ≈ 0.72–0.73), suggesting a cooperative but slightly reduced interaction at increased doses. These findings indicate that the DZN and PNR combination enhances anti-proliferative effects in BGC-823 cells, with the most substantial synergy observed at lower concentrations. To further our understanding of their effects at the molecular level, we decided to continue utilizing the following ratios (1). DZN and PNR, each at 10 µM; (2). DZN and PNR at 20 µM; (3). At 30 µM, DZN and PNR.

**Table 1:** Combination index values for DZN and PNR (1:1)

Concentrations	CI Value	CI Effect
10µM	0.52345	Synergistic
20µM	0.56543	Synergistic
30µM	0.57653	Synergistic
40µM	0.71984	Modest Synergistic
50µM	0.73498	Modest Synergistic

The combination index (CI) values for DZN and PNR at a 1:1 ratio were calculated to assess their interaction effects on BGC-823 cell viability. The results demonstrated a dose-dependent trend, where CI values ranged from 0.52345 to 0.73498 across different concentrations (Table/Figure X). At lower concentrations (10–30 µM), the combination exhibited a synergistic effect (CI < 1), indicating enhanced inhibitory activity compared to individual treatments. At higher concentrations (40–50 µM), the effect remained modestly synergistic (CI ≈ 0.72–0.73), suggesting a cooperative but slightly reduced interaction at increased doses. These findings indicate that the DZN and PNR combination enhances anti-proliferative effects in BGC-823 cells, with the most substantial synergy observed at lower concentrations.”

### Cell proliferation assay by edu labelling

**Fig. 2:**
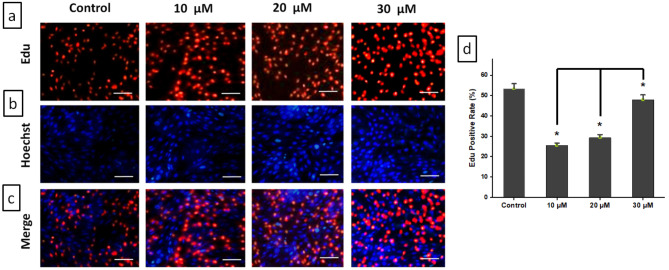
Impact of the combination of DZN and PNR on apoptosis. (**a**) EdU-based cell proliferation test. (**b**) BGC-823 cells treated with varying concentrations of DZN + PNR for a 24-hour period were stained with Hoechst 33,258. Bright-blue fluorescence and highly dispersed or compacted nuclei (at 100× magnification) were indicative of apoptotic cells. (**c**) Combine the EdU and Hoechst 33,258 images. Data are represented as mean ± SD. *P < 0.05 compared with control group, *n* = 3. Scale bar: 50 μm

The effects of DZN + PNR therapy on the proliferation of BGC-823 cells were assessed using the EdU DNA cell proliferation assay. As anticipated for a population that divides quickly, a 2 h pulse of EdU labeled around one-third of the control cells (Figs. 2a), but only roughly one-fourth of the cells transfected with the combination of DZN + PNR (Figs. 2a). The findings showed that the combined use of 30 µM DZN and PNR considerably reduced the rate of BGC-823 cell growth.

### Synergistic induction of apoptosis by co-treatment with DZN and PNR in BGC-823 cells

Using a fluorescent microscope, Hoechst staining was employed to evaluate the nuclear morphological alterations in BGC-823 cells. The control group’s round nuclei were uniformly stained blue, as demonstrated by the Hoechst staining results (Fig. 2b). In cell death situations, significantly enhanced chromatin condensation or fragmentation is seen in brilliant blue. Comparing DZN and PNR-treated cells to untreated cells, the former displayed significantly stronger fluorescence and compacted nuclei.

### Impact of DZN and PNR on apoptotic measures using the AO/EB staining assay in BGC-823 cells

**Fig. 3:**
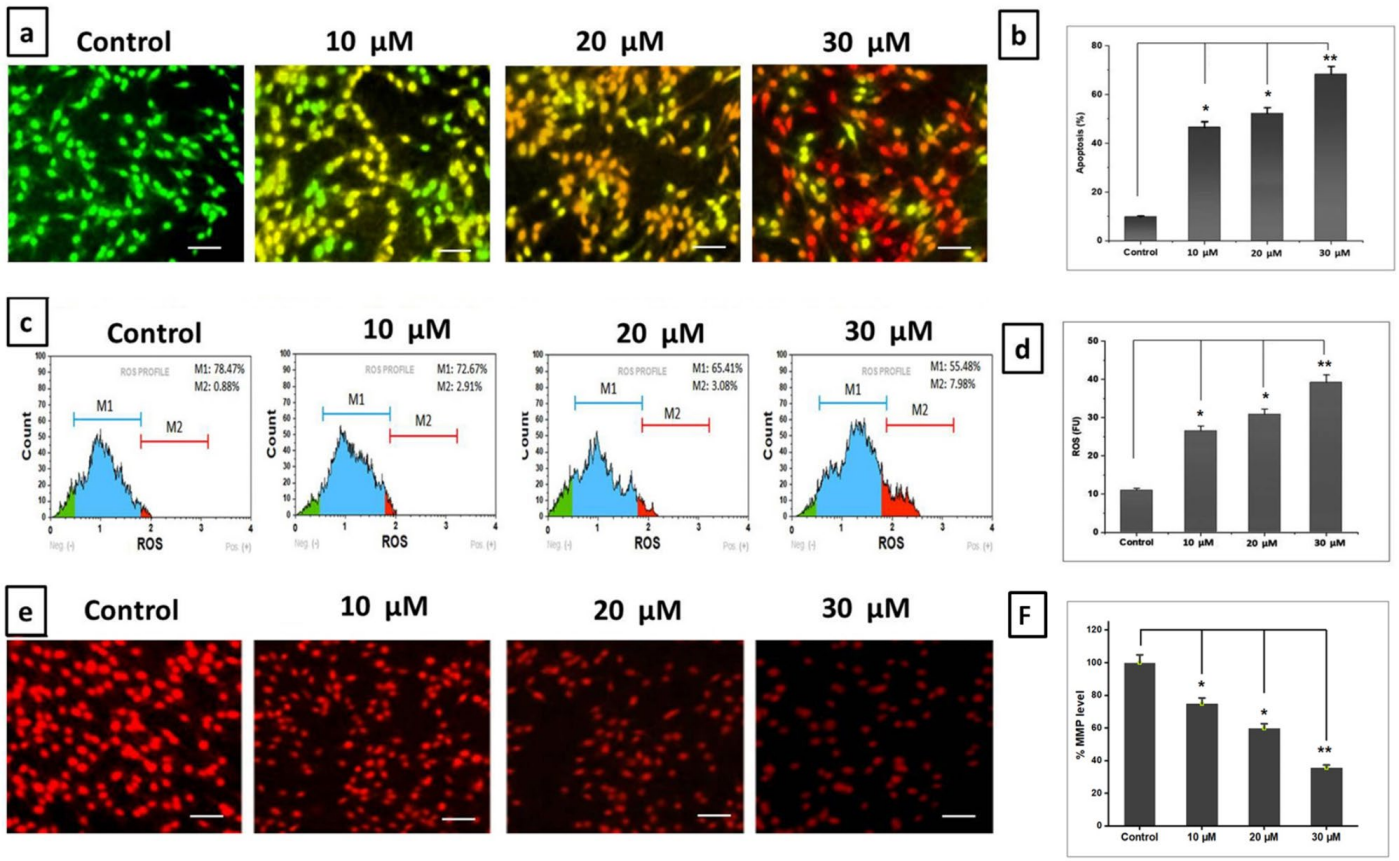
(**a**) Furthermore, the cells were stained with AO/EtBr in an effort to identify the apoptotic stage utilizing a combination of DZN and PNR. As per an alternative proposition, an amalgamation DNN + PNR therapy procedure resulted in more robust apoptosis, as the investigation’s findings showed. (**b**) The bar graph represents the percentage of cells that underwent apoptosis in the control, 10, 20, and 30 µM DZN and PNR treatments. Every value in the above data is the mean ± standard deviation of three separate studies, with ∗ *P* < 0.05, ∗∗ *P* < 0.01 (**c**) The generation of ROS in BGC-823 cells was triggered by the combined action of DZN and PNR. Using a flow cytometer, the amount of intracellular ROS buildup was quantified and stained with DCFH-DA. (**d**) Comprehensive evaluation of ROS levels inside cells. (**e**) The impact of DZN and PNR combo on MMP levels in BGC-823 cells. Results from Rh-123 staining indicate revealed the cells supplemented with combination of DZN and PNR had lower MMP levels compared the untreated group. The aforementioned results are all the mean ± standard deviation of three separate studies, with ∗ *P* < 0.05 and ∗∗ *P* < 0.01 vs. control. Scale bar: 50 μm

Most of the time, the nuclei were observed to have broken up into smaller pieces, indicating that BGC-8230 cells treated with 10, 20, and 30 µM of DZN and PNR were forming apoptotic bodies. The administration of 30µM PNR and DZN combined led to an upsurge in the quantity of late apoptotic cells, which were identified by coloring the cells an orange red-color, indicating that the membranes had been destroyed (Fig. 3a). The unaltered control cells showed stained nuclei with undamaged cell structure; these changes were not investigated in these cells. Consequently, our results indicated that the DZN + PNR combination strongly affects nuclear anatomy, which is closely linked to apoptosis. At the different doses (10, 20, and 30 µM/mL) of the DZN and PNR-treated groups, the ratio of apoptotic cells was 52.45, 46.23, and 68.46%, respectively (∗ *P* < 0.05 and ∗∗ *P* < 0.01) (Fig. 3b).

### Impact of DZN and PNR on the generation of ROS in BGC-823 cells

Apoptosis must be induced by elevated ROS production. Flow cytometry was used to determine the intracellular ROS levels following treatment with various DZN and PNR concentrations of substances. By using flow cytometry, we discovered that 10, 20, and 30 µM of DZN and PNR effectively increased the ROS formation in BGC-823 cells (Fig. 3c). The ROS levels of BGC-823 cells were 1.9, 2.5, and 19.5 times higher than those of the control group following exposure to10, 20, and 30 µM DZN and PNR (∗ *P* < 0.05) (Fig. 3d). Thus, it was evident from the results of flow cytometry that the combined use of DZN and PNR therapy resulted to enhanced ROS generation in BGC-823 cells.

### DZN and PNR’s impact on MMP damage in BGC-823 cells

The mitochondrial membrane potential, or MMP, is an indicator of the cell’s physiological state. Additionally, it serves as a crucial indicator of the stability and decrease of the mitochondrial membrane, which are earlier processes that lead to apoptosis. Cells were used for rhodamine 123 dye staining to evaluate the MMP reduction in combination with 10, 20, and 30 µM DZN and PNR treated BGC-823 (Fig. 3e). Increased red fluorescence reflection, indicative of a polarized mitochondrial cell membrane, is released by control cells. On the other hand, BGC-823 cells treated with DZN and PNR (10, 20, and 30 µM) showed significantly changed mitochondrial membrane potential, which consistently reduced the emission of red fluorescence. The treatment affected the MMP and resulted in an early apoptotic signal.

### The synergistic effect of DZN and PNR caused cell cycle arrest in BGC-823 cells

**Fig. 4:**
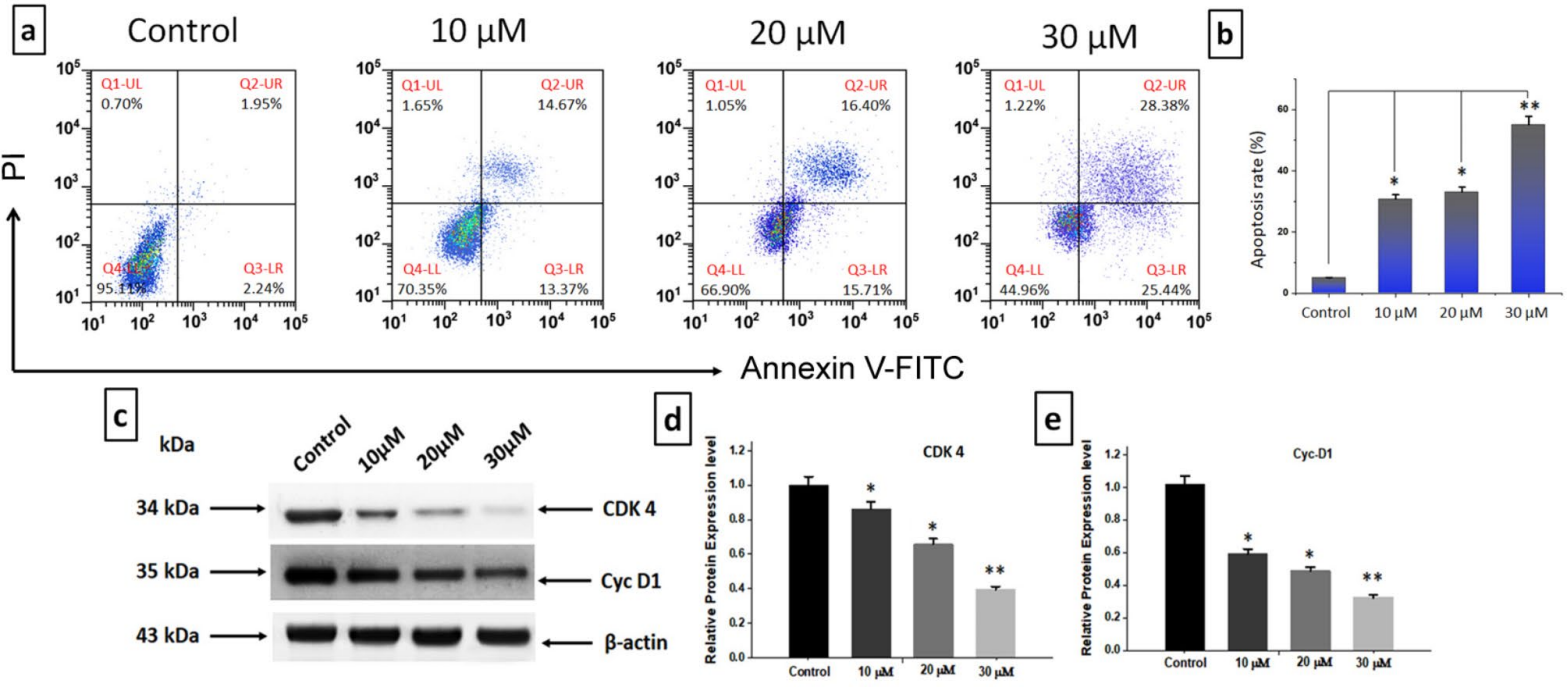
The combined effects of DZN and PNR on the cell cycle. (**a**) BGC-823 cells treated for 24 h with 10, 20, and 30µM DZN and PNR. PI labeling was used in flow cytometry to assess the cell cycle. (**b**) The proportion of cells that are spread throughout the cell cycle. (**c**, **d**, **e**) Western blotting was used to determine the amounts of CDK4 and cleaved CyclinD1. The data are expressed as mean ± S.D. from three independent experiments. *p < 0.05 and **p < 0.01 vs. control

After subjecting BGC-823 cells to 10, 20, or 30 µM of DZN + PNR for a whole day, the cell cycle arrest was examined using flow cytometry to investigate the impact of these two compounds on the cell cycle. According to the results, BGC-823 cells were significantly arrested in the G0/G1 phase when combined with PNR and DZN. This effect was even more pronounced in the groups that received both treatments together (**p < 0.01) (Fig. 4a, b). This suggests that PNR and DZN together inhibited cell growth by blocking the cell cycle. Western blotting analysis subsequently assessed the expression levels of cell-cycle-related proteins to investigate the possible molecular mechanism driving G0/G1 cell cycle arrest (Figs. 4c). As seen in Figs. 4d, e, CyclinD1 and CDK4 expression levels were lowered following the combination of DZN and PNR therapy, and CyclinD1 and CDK4 expression was further lowered (***p < 0.001) in BGC-823 cells following the concentration-based combined therapy.

### Impact of DZN and PNR on apoptosis and inhibited intrinsic expression of STAT3 in BGC-823cells

**Fig. 5:**
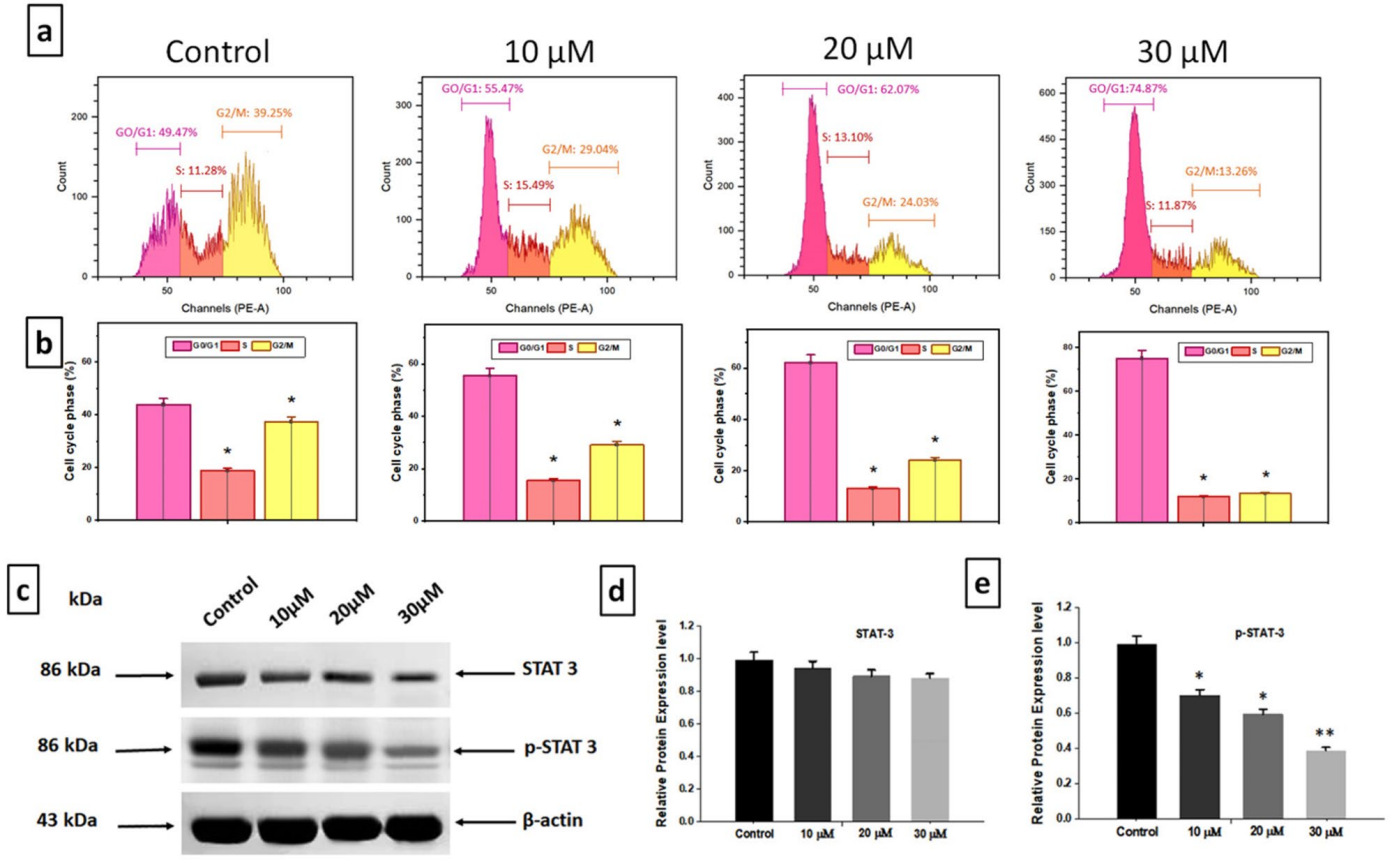
In BGC-823 cells, the simultaneous administration of DZN and PNR caused apoptosis and inhibited the constant activation of STAT3. (**a**) Using a combo of DZN and PNR, BGC-823 cells were cultured for 24 h. (**b**) Statistical evaluation of assays for cell apoptosis. The mean ± standard deviation of three separate experiments is shown for all of the information provided above. (**c**) For a duration of 24 h, BGC-823 cells were cultured in a mixture of 10, 20, and 30µM DZN and PNR. STAT3 and p-STAT3 were found using western blotting (Tyr705). An internal control was employed, namely β-Actin. (**d**, **e**) Statistical evaluation of STAT3 and p-STAT3 respective protein expression rates. All of the following results are shown as the mean ± standard deviation of three separate studies, with ∗ *P* < 0.05, ∗∗ *P* < 0.01 vs. control

A crucial method by which anticancer drugs work is their induction of apoptosis. Thus, we looked into the possibility that DZN and PNR’s cytotoxicity was linked to the BGC-823 cells’ activation of apoptosis. The findings of the flow cytometry assay showed that BGC-823 cell death could be induced by 10, 20, and 30 µM of DZN and PNR, as shown in Fig. 5a. The outcomes of the flow cytometry-detected apoptosis showed that following incubation with varying concentrations of 10, 20, and 30 µM of DZN and PNR, the apoptotic cell count rose from 4.89 to 29.69%, 33.16%, and 55.04% (Fig. 5b). According to the aforementioned clinical results, DZN + PNR together inhibited the growth of tumor cells by causing ROS generation, cell death, and cell cycle arrest. Further research is still necessary to understand the fundamental principles that explain how the combined use of DZN and PNR affects these cellular functions.

The development and spread of cancers have been linked to aberrant STAT3 activation in gastric tumor cells. To control the activation of constitutive STAT3, the potential of DZN and PNR was investigated further. Western blotting experiments demonstrated that phosphorylated STAT3 expression was lowered in a dose-dependent manner, while STAT3 levels remained unchanged. As shown by Fig. 5c, d, e, the constitutive phosphorylation of STAT3 in BGC-823 cells was decreased by the combined usage of DZN and PNR (10, 20, 30 µM). DZN and PNR were found to be potential STAT3 inhibitors overall.

### Synergistic effect of DZN and PNR inhibited STAT3 pathway

**Fig. 6:**
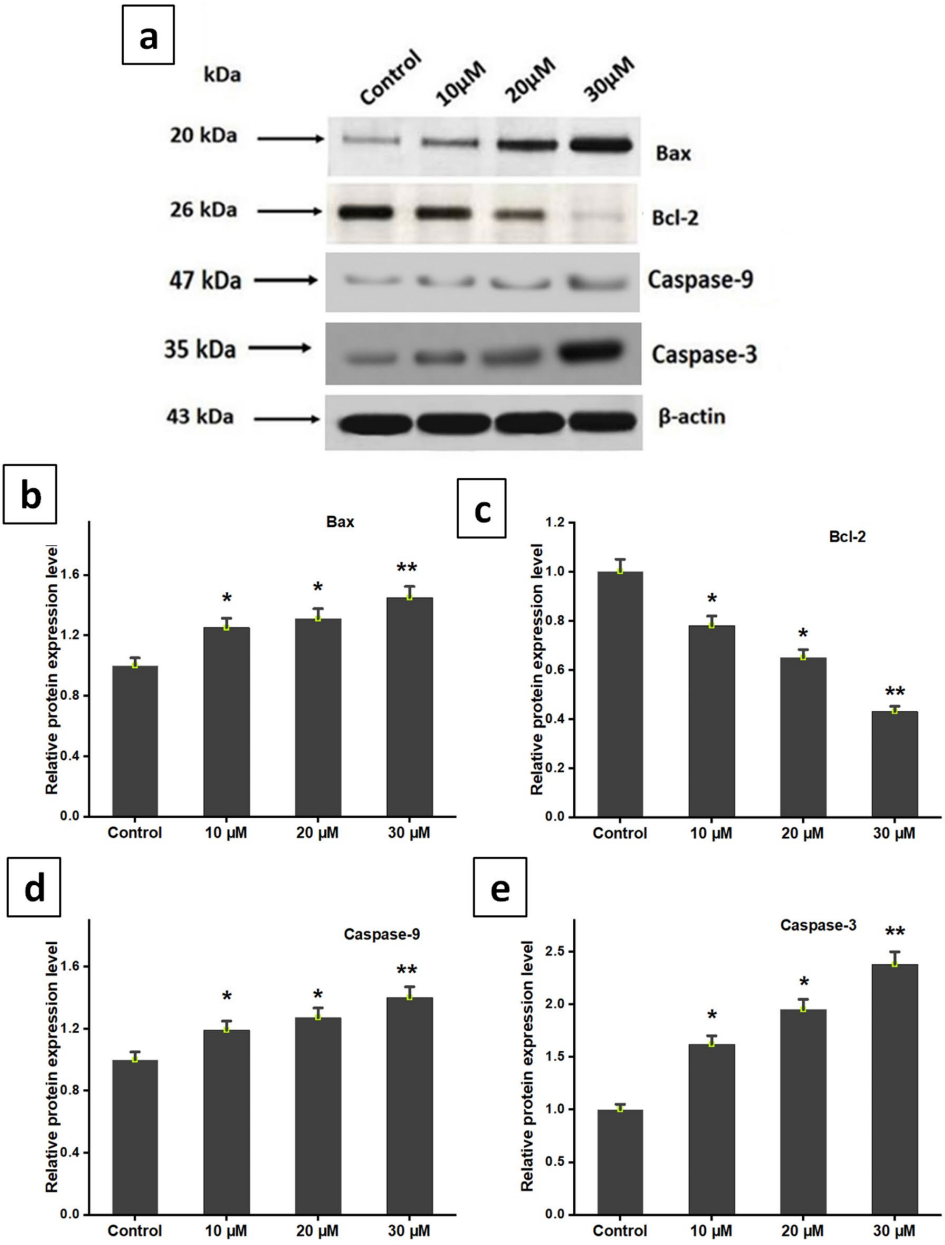
DZN and PNR together directly inhibited BGC-823 cell proliferation by targeting STAT3. (**a**) DZN and PNR were cultivated in combination in BGC-823 cells for a whole day. Using the western blotting technique, the levels of many proteins (Bax, Bcl-2, Caspase 9, and Caspase 3) were determined. (**b-e**) Using Image Studio, the densitometric evaluation of the previously mentioned proteins was evaluated. The equal loading of proteins was confirmed by the replication of the housekeeping gene, β-actin. All of the following results are shown as the mean ± standard deviation of three separate studies, with ∗ *P* < 0.05, ∗∗ *P* < 0.01 vs. control

There has been evidence that the active STAT3 signaling pathway can control the expression of associated proteins, such as those involved in angiogenesis, cell proliferation, and survival. It became vital to investigate if DZN and PNR had an impact on the development of decreased proteins of the STAT3 signaling pathway since their synergistic action interacted with the phosphorylation of STAT3 (Fig. 6). When the cell cycle progresses from the growth and synthesis stage, cyclin D1 is an essential component. In cells treated with a combination of DZN and PNR, cyclin D1 expression levels dramatically dropped, as demonstrated by western blotting. It was discovered that the expression of Bcl-2, decreased in a dose-related (10, 20, 30 µM) way. As DZN and PNR dosages increased concurrently, so did the expression of the apoptotic protein Bax (Fig. 6a-e). Our findings confirm the well-established relationship between STAT3 and tumor cell death, specifically through caspase-3 and caspase-9 activation. According to Western blotting, the result indicates increased expression levels of caspases while treated with DZN and PNR, further supporting this link (Fig. 6f, g, h). The findings above unambiguously demonstrated that DZN and PNR (30µM) together suppressed cell proliferation (*p < 0.05) by controlling the STAT3-mediated apoptotic pathway. Downregulation of STAT3 activity, induces apoptosis by caspases activation.

### Combination of DZN and PNR impeded the migration of gastric cancer cells and the FAK signaling pathway

**Fig. 7:**
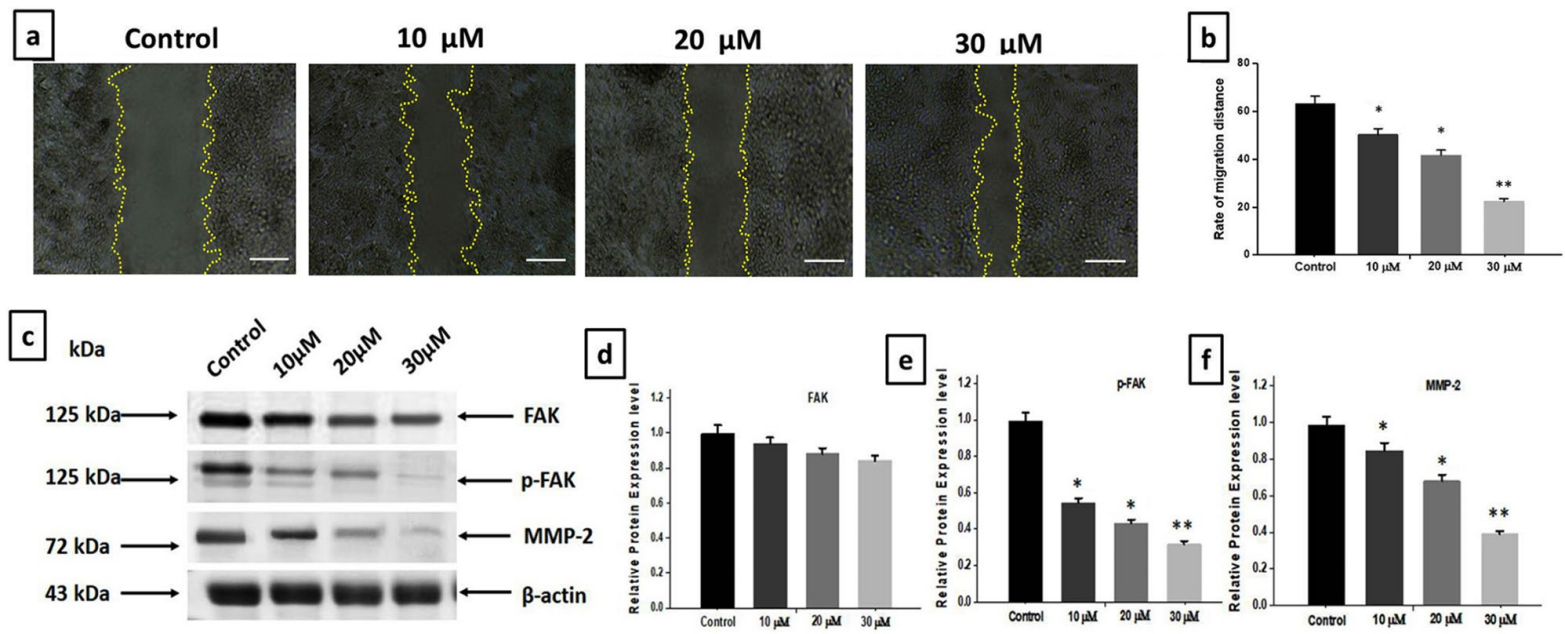
In BGC-823 cells, DZN and PNR blocked the FAK signaling pathway and cell migration. (**a**) To determine the impact of combined DZN and PNR usage on migration in scratched wounds, a wound healing test was conducted. (**b**) By counting three wound healing sites at random in each image, a statistical analysis of the cell migration rate is performed. The information mentioned above is all the mean ± standard deviation from three different experiments. Scale bar: 50 μm. (**c**) DZN and PNR were combined and incubated with BGC-823 cells for 24 h at varying concentrations. Western blotting was used to assess the expression of p-FAK (Tyr397) and MMP-2. A loading control was applied using β-Actin. (**d-f**) Analytical evaluation of FAK, p-FAK (Tyr397), and MMP-2 differential expression rates. All of the following results are shown as the mean ± standard deviation of three separate studies, with ∗ *P* < 0.05, ∗∗ *P* < 0.01 vs. control

A wound-healing assay was performed to evaluate the effects of DZN and PNR on BGC-823 cell migration. The wound healing assay (Fig. 7a) shows that in control cells exhibited a high migration rate, gastric cancer cell migration was progressively inhibited with increasing concentrations of DZN and PNR (10, 20, and 30 µM) at 24-hour incubation. Quantification of migration percentages (Fig. 7b) demonstrated a significant dose-dependent reduction in cell migration, with migration rates of approximately 60.2% (control), 51.5% (10 µM), 38.9% (20 µM), and 21.5% (30 µM) (*p* < 0.05). These findings indicate that DZN and PNR effectively suppress BGC-823 cell migration. To further investigate the anti-metastatic potential of DZN and PNR, we examined the expression of FAK, p-FAK (Tyr397), and MMP-2 using Western blot analysis (Fig. 7c). The results showed that total FAK expression remained relatively unchanged (Fig. 7d), while the levels of phosphorylated FAK (p-FAK (Tyr397), Fig. 7e) and MMP-2 (Fig. 7f) were significantly reduced in a dose-dependent manner. These findings suggest that the combination of DZN and PNR inhibits cancer cell migration by targeting the FAK/STAT3 signaling pathway and decreasing the expression of metastatic markers.

## Discussion

One of the key ongoing issues in gastric cancer treatment is patient progression. Finding new active compounds is required because the majority of chemotherapeutic medicines used in clinical practice to treat stomach cancer are carcinogenic and highly toxic, and they are ineffective when the illness has progressed [27]. Therefore, it makes sense to look into ways to suppress the growth of gastric cancer cells while minimizing negative effects for patients. According to current information, DZN and PNR are harmless to human normal cells and may have some anticancer activities [23, 28].

As members of the isoflavone family, daudzein and puerarin are readily obtained through diet, particularly from soy products or soybeans and Radix *pueraria lobate*. Several malignancies have been examined using DZN and PNR because of their anti-proliferation properties [22, 23, 29]. In this investigation, we conducted the experiments using a combination of DZN and PNR (10, 20, and 30 µM). When associated with the untreated cells, the combined effects of DZN and PNR on apoptosis induction, cell cycle arrest, and invasion prevention in BGC-823 cells are noteworthy. Furthermore, co-treatment with PNR and DZN regulated the impairment of the FAK and STAT3 pathways in a synergistic manner. According to these findings, DZN and PNR together have potent anti-gastric cancer properties.

The results of this investigation showed how DZN and PNR affected the growth of gastric cancer cells in vitro. The results of the investigation demonstrated that DZN and PNR together reduced BGC823 cell viability in a concentration-based way. The GES-1 normal gastric cell viability did not significantly alter after receiving DZN and PNR therapy. These results demonstrated that the combined use of DZN and PNR effectively reduced the viability of gastric cancer cells. These characteristics align with those of second-generation therapies for cancer, which show relatively moderate toxicity to normal cells but specific cytotoxicity on cancerous cells [30].

As widely recognized, apoptosis and the suppression of proliferation go hand in hand. The process of apoptosis is essential for the death of cancer cells. Thus, in the majority of cancer therapies, apoptosis has emerged as the primary indication. Apoptosis in a variety of cancer cells was previously reported to be induced by DZN and PNR [31–36] Additionally, we were able to confirm that DZN plus PNR can dramatically enhance the proportion of apoptotic cells using annexin V-FITC/propidium iodide (PI) labeling. The apoptotic molecule Bax, which is a component of the apoptotic process, was also expressed at an increased level. There was a downregulation of the Bcl-2 protein. According to [37], puerarin therapy enhances the expression level of Bax and diminutions the expression of c-myc and Bcl-2 in colon cancer HT-29 cells while reducing cell proliferation.

Numerous previous studies have demonstrated that the inhibition of cancer cell proliferation is associated with cell cycle arrest [38, 39]. In our study, we observed that the combined treatment with DZN and PNR induced apoptosis and led to G0/G1 phase cell cycle arrest in BGC-823 cells. Specifically, our results showed a downregulation of CDK-4 and cyclin D1, key regulators of the G0/G1 phase transition, confirming the cell cycle arrest effect. Additionally, our findings revealed an increase in caspase-3 and caspase-9 expression following DZN and PNR treatment, supporting the induction of apoptosis. These results provide strong evidence that the combination of DZN and PNR effectively inhibits gastric cancer cell proliferation by triggering both cell cycle arrest and apoptotic pathways.

Previous studies have reported that daidzein induces G1 and G2/M phase arrest in human breast carcinoma cells and G1 phase arrest in human melanoma cells [40, 41], while puerarin has been shown to inhibit glioblastoma cells, and breast cancer cell proliferation by inducing apoptosis and G2/M phase arrest [21, 42]. Furthermore, Gan and Yin [43] found that puerarin causes G1 phase arrest in mantle cell lymphoma cells. While previous studies have explored the individual cytotoxic effects of DZN and PNR in cancer, our study is the first to elucidate their combined impact on gastric cancer cells, highlighting their potential synergistic therapeutic effects. Thus, our findings provide novel insight into the synergistic effect of DZN and PNR in inducing G0/G1 phase arrest and apoptosis in BGC-823 cells, supporting their potential therapeutic application in gastric cancer.

According to recent studies, DZN hinders cancer cell progression by activating the MAPK family signaling pathway [44]. Additionally, Zheng et al. [22] reported that daidzein reduces ERK phosphorylation in cancer cells, while puerarin influences bladder cancer cell survival and induces apoptosis via the mTOR/p70S6K signaling pathway [23]. However, the effect of DZN and PNR on the proliferation, migration, and invasion of gastric cancer cells remains unclear.

In this study, we provide the first evidence that the combination of DZN and PNR significantly inhibits gastric cancer cell migration and invasion by downregulating STAT3/FAK signaling and suppressing MMP-2 expression. These findings are consistent with our wound healing assay results, which demonstrate a dose-dependent reduction in cell migration, and our Western blot analysis, which confirms a decrease in p-FAK (Tyr397) and MMP-2 expression. Together, these results support the conclusion that DZN and PNR work synergistically to suppress the metastatic potential of BGC-823 cells.

MMPs play a critical role in gastric cancer metastasis by degrading the extracellular matrix, facilitating tumor cell dissemination. A previous study reported that cisplatin and berberine inhibit the MMP-2 and MMP-9 pathways, reducing cell proliferation and inducing apoptosis in lung cancer [45]. In line with this, our study demonstrates that DZN and PNR significantly suppress BGC-823 cell migration and invasion by downregulating MMP-2 expression, further supporting their potential as anti-metastatic agents.

In order to clarify the molecular process that occurs when DZN and PNR together inhibit the migration and invasion of cancer cells, we looked into STAT3/FAK signaling as a possible target for medication. Because it increases MMP activity, elevated FAK phosphorylation is closely correlated with the acquisition of glioblastoma cell migration and proliferation [46]. For example, in tumor growth and dissemination, STAT3 activation controls FAK phosphorylation and MMP-2 activity [47]. Furthermore, no study has shown that blocking STAT3 signaling in DZN + PNR affects the invasion, and development of gastric cancer cells.

Through the inhibition of FAK phosphorylation, the combination of DZN + PNR limits cell invasion as demonstrated by our findings. Furthermore, the combined effects of DZN and PNR markedly reduced cancer cell migration and STAT3 phosphorylation. Decreased phospho-STAT3-driven transcription is the outcome of DZN and PNR’s effective downregulation of phospho-STAT3. Numerous studies have shown constitutive activation of STATs, in particular STAT3, in a variety of human tumor cell lines and malignancies. STAT3 is now recognized as a significant participant in the development of human cancer. Therefore, because of their expected efficacy against cancer, STAT3 signaling blockers are of tremendous interest [6]. These results indicate that DZN and PNR effectively inhibit gastric cancer cell migration and invasion, likely through suppression of the FAK/p-FAK signaling pathway and MMP-2 expression. The findings suggest that targeting FAK signaling with DZN and PNR may serve as a potential strategy for limiting gastric cancer metastasis.

The combination of Daidzein (DZN) and Puerarin (PNR) exhibits potential as an anti-cancer treatment agent, especially for gastric cancer, according to our findings and the body of current research. Our study showed that the combination of DZN and PNR reduced the levels of STAT3 phosphorylation in a concentration-dependent way. This is consistent with earlier research showing that STAT3 plays a negative regulatory role in the development of gastric cancer [48]. Furthermore, the expression of downstream STAT3 target proteins, such as cyclin D1 and Bcl-2, which are essential for controlling apoptosis and cell cycle progression, was altered by the combination of DZN and PNR. This alteration is in line with research showing that DZN inhibits the production of cyclin D1 and Bcl-2, which causes cancer cells to undergo cell cycle arrest and apoptosis [22].

Additionally, by raising levels of pro-apoptotic proteins including Bax, caspase-9, and caspase-3 and lowering levels of anti-apoptotic Bcl-2, DZN and PNR jointly affected the apoptotic pathway. This supports research showing that DZN administration raised Bax and activated caspase-9 and caspase-3 in melanoma cells, indicating that the combination triggers apoptosis via the intrinsic mitochondrial route [49]. Notably, our research offers the first proof that DZN and PNR together operate as an anti-cancer drug by blocking MMP-2 production in human gastric cancer cells through the STAT3/FAK signalling pathways. Given that MMP-2 is known to promote tumour invasion and metastasis, this discovery is noteworthy [48]. All of these findings point to the possibility that using DZN and PNR to target the STAT3 signalling pathway could be a successful therapy or preventative approach for gastric cancer.

## Conclusion

This study provides evidence that the combination of DZN and PNR effectively inhibits the proliferation of gastric cancer (GC) cells by targeting the STAT3/FAK signaling pathway. Our findings demonstrate that DZN and PNR suppress STAT3 and FAK phosphorylation, leading to the downregulation of key metastatic and survival markers, including cyclin-D1, Bcl-2, and MMP-2. Furthermore, the combination treatment induces apoptosis and disrupts the cell cycle, contributing to the inhibition of cancer cell growth. Overall, these results highlight the therapeutic potential of DZN and PNR as natural compounds for gastric cancer treatment. Further research is warranted to explore their clinical applications and the molecular mechanisms underlying their anti-cancer effects.

## Data Availability

No datasets were generated or analysed during the current study.

## Abbreviations

DZN: Daidzein
PNR: Puerarin
GC: Gastric cancer
STAT3: Signal transducer and activator of transcription 3
FAK: Focal adhesion kinase
JAKs: Janus kinases
MMP: Mitochondrial membrane potential
